# From Challenges to Solutions: Patient Insights Inform Psychological Intervention Design After Spontaneous Coronary Artery Dissection

**DOI:** 10.1016/j.jacadv.2026.102792

**Published:** 2026-06-17

**Authors:** Lisa-Marie Maukel, Thais Coutinho, Sharon Mulvagh, Mina Madan, Christine Pacheco, Karen Bouchard, Derek So, Jennifer Reed, Shuangbo Liu, Louise Sun, Amélie Paquin, Nadia Lappa, Jacqueline Saw, Heather Tulloch

**Affiliations:** a University of Ottawa Heart Institute, Ottawa, Ontario, Canada; b Mayo Clinic, Rochester, Minnesota, USA; c Dalhousie University, Halifax, Nova Scotia, Canada; d Sunnybrook Health Sciences Centre, University of Toronto, Toronto, Ontario, Canada; e Université de Montréal, Montréal, Quebec, Canada; f University of Ottawa, Ottawa, Ontario, Canada; g University of Manitoba, Winnipeg, Manitoba, Canada; h Stanford University School of Medicine, Stanford, California, USA; i Department of Cardiology, Quebec Heart and Lung Institute – Laval University, Quebec City, Quebec, Canada; j Patient partner of the University of Ottawa Heart Institute, Ottawa, Ontario, Canada; k University of British Columbia, Vancouver, British Columbia, Canada

**Keywords:** mental health, psychological intervention needs, SCAD, sex differences, spontaneous coronary artery dissection

## Abstract

**Background:**

Spontaneous coronary artery dissection (SCAD) is a cause of acute coronary syndrome, primarily affecting women. SCAD is associated with elevated psychological distress and cardiac rehabilitation programs rarely meet these patients’ mental health needs. Although the challenges faced by SCAD survivors are well documented, evidence on *how* patients want them to be addressed remains limited.

**Objectives:**

This study aimed to examine psychological intervention preferences among SCAD survivors, including potential sex differences.

**Methods:**

Patients diagnosed with SCAD within the past 3 years were recruited from 6 Canadian hospitals. Participants completed questionnaires assessing sociodemographic and clinical characteristics, and rated their interest in intervention content and delivery methods using a 5-point Likert scale. Descriptive statistics were calculated and sex differences were analyzed using *t*-tests.

**Results:**

Among 326 participants (92.9% female; mean age 53.3 ± 11.1 years) top-rated intervention components included stress management (3.7 ± 1.2; 64.5% rated very/extremely interested), relaxation techniques (3.5 ± 1.2; 55.4%), cognitive behavioral strategies (3.5 ± 1.3; 56.8%), and processing the SCAD-related trauma (3.4 ± 1.4; 53.5%). Patients preferred SCAD-specific programming (3.9 ± 1.1; 70.8%) delivered by health care professional (3.4 ± 1.3; 56.2%) offered within 1 to 3 months postevent (3.5 ± 1.4; 61.8%). Women expressed greater interest than men in topics such as health advocacy and communication with children (all *P* < 0.05). Many women (55.1%) also conveyed high interest in gender-specific interventions.

**Conclusions:**

SCAD survivors prefer early, individualized, SCAD- and gender-specific interventions focusing on cognitive, emotional, and trauma processing, delivered by health care professionals. Psychological interventions targeting these elements need to be developed and tested for SCAD survivors.

Spontaneous coronary artery dissection (SCAD) is an increasingly recognized cause of acute coronary syndrome (ACS), disproportionately affecting women (∼90%).^1^ It is the leading cause of ACS during pregnancy and accounts for up to 35% of ACS cases among women under 50 years of age.^2^ SCAD is characterized by the formation of an intramural hematoma in 1 or more epicardial coronary arteries, sometimes accompanied by an intimal tear. Unlike other forms of ACS, SCAD often occurs in the absence of atherosclerosis, trauma, iatrogenic causes,^3^ or traditional cardiovascular risk factors.^4^

Despite a relatively low mortality rate of approximately 1% over 33 months,^5^ SCAD has significant long-term consequences, including recurrence rates of up to 10% in the first 3 years postevent.^6^, ^7^, ^8^ The condition’s unpredictable course, substantial somatic symptom burden (eg, persistent chest pain), and the lack of evidence-based disease-specific secondary prevention strategies^9^ contribute to considerable emotional and psychological burden.

Psychological distress is highly prevalent among individuals with SCAD. Over 20% reported moderate to severe depression or anxiety symptoms, whereas post-traumatic stress symptoms occurs in up to one-third of patients.^10^, ^11^, ^12^ Cardiac-specific anxiety—marked by hypervigilance to cardiac sensations, fear of recurrence, and avoidance—affects more than 50%,^12^ exceeding rates in other cardiac populations (20% to 30%).^13^^,^^14^ This distress is reported more frequently by women, may occur even in individuals without prior mental health conditions, and often persists for years.^12^ These findings are particularly concerning, as psychological distress has been associated with a 30 to 60% increased risk of adverse cardiovascular outcomes.^15^

Not surprisingly, then, the American Heart Association, health care providers, and patients with SCAD emphasize the need for SCAD-specific mental health interventions to complement traditional cardiac rehabilitation (CR).^2^^,^^16^^,^^17^ Existing SCAD-tailored mental health interventions have produced limited benefits; results from the 3 small proof-of-concept intervention studies (Ns = 7-33) did not produce clinical significance changes in depression and anxiety symptoms. In addition, all 3 studies faced retention challenges,^18^, ^19^, ^20^ with dropout rates as high as 48%.^20^

Although extensive research has clarified the mental health and psychosocial challenges faced by SCAD patients,^1^^,^^11^^,^^12^^,^^21^, ^22^, ^23^, ^24^, ^25^, ^26^, ^27^, ^28^, ^29^, ^30^, ^31^ there remains a lack evidence on *how* to effectively address these needs, particularly from the patient perspective. Qualitative feedback from the early interventions indicated that patients valued support in understanding and accepting their emotions,^18^ with mixed views on mindfulness techniques.^18^^,^^20^ To date, no study has systematically investigated the intervention components (eg, psychoeducation, cognitive-behavioral techniques, and relaxation) and delivery formats (eg, group vs individual, frequency) patients with SCAD consider most desired to address their mental health. The present study fills this gap using a codesign patient engagement framework that emphasizes structured collaboration between patients and health care professionals to jointly identify, implement, and refine improvements to care.^32^^,^^33^ Following the obesity-related behavioral intervention trials model for psychological and behavioral intervention development,^34^ this study is the first large-scale effort to systematically identify SCAD survivors’ preferences for psychological intervention content and format and examine potential sex differences in these preferences.

## Methods

### Study population and recruitment

Individuals with an angiography-confirmed diagnosis of SCAD within the last 3 years were recruited from 6 Canadian hospitals from April 2022 to January 2025. Inclusion criteria included age ≥18 years, fluency in English or French, and the ability to provide informed consent. Participants were recruited through hospital-based cardiology services. The research coordinator informed eligible participants about the study and obtained informed consent. Participants had the option to complete the study questionnaires either on paper (on-site or at home using a preaddressed stamped envelope) or electronically via the secure REDCap platform.^35^ This study was approved by the Ottawa Health Science Network Research Ethics Board (Protocol ID: 20210797-01H).

## Measurements

### Demographic and medical information

Participants completed a standardized questionnaire capturing demographic data (eg, age, sex, and ethnicity), physical health conditions, and mental health history. Self-reported medical information was verified with participants’ medical records.

### Intervention preferences

Participants rated the perceived importance of 32 potential intervention components, informed by previous research,^17^ using a 5-point Likert scale (1 = not at all important; 5 = extremely important). These components included evidence-based psychological strategies such as psychoeducation (eg, sleep hygiene) and cognitive-behavioral techniques. Practical support options (eg, vocational counseling) were also assessed. Using a similar scale (1 = not at all interested; 5 = extremely interested), participants rated their interest in various delivery formats (eg, online vs in-person delivery, group vs individual) and timing. Open-ended questions allowed participants to suggest additional content or delivery preferences.

### Statistical analysis

All analyses were conducted using IBM SPSS Statistics (version 29). Descriptive statistics were calculated for demographic, clinical, and intervention preference-related variables. Continuous variables were summarized using mean and SD, whereas categorical variables were reported as frequencies and percentages. Sex differences were analyzed using Student’s or Welch *t*-tests, depending on variance equality.

We acknowledge that factors other than sex may influence intervention preferences; however, given the low number of male participants (n = 23), adjusted analyses were not feasible. We therefore conducted exploratory analyses.

To assess potential differences in intervention preference based on time since the SCAD event, exploratory descriptive analyses compared mean scores for preferences across 4 categories: 0 to 3 months, 4 to 12 months, 13 to 24 months, and 25+ months post-SCAD.

We also conducted descriptive comparisons of the mean intervention preference scores between participants with pregnancy-related SCAD and those with non–pregnancy-related SCAD.

In an exploratory multiple regression of intervention content preferences, we included sex, physical burden as measured by the Seattle Angina Questionnaire^36^ subscale symptom frequency (higher scores indicating chest pain symptoms), and mental health status. Mental health status was defined as clinically significant anxiety or depressive symptoms, indicated by a score ≥10 on either the Generalized Anxiety Disorder-7 or the Patient Health Questionnaire-9.^37^^,^^38^

Because 22 patients had a time since diagnosis between 36 and 48 months, exceeding the inclusion criterion, analyses were performed both with and without them. As no meaningful differences were found, these patients were retained to preserve statistical power. Statistical significance was set at *P* ≤ 0.05.

## Results

### Demographic and clinical characteristics

The study included 326 patients with SCAD (92.9% female; mean score [*M*] age = 53.3 ± 11.1 years) (Table 1). Most patients completed postsecondary education (81.3%), were in a partnership (73.3%), and 53.0% had children living with them. The most common clinical presentation was non–ST-segment elevation myocardial infarction (65.0%), followed by ST-segment elevation myocardial infarction (30.6%), with SCAD type II as the most frequent SCAD classification observed (48.2%). The mean time from diagnosis to study enrollment was 15.9 ± 12.5 months.

**Table 1:** Demographic and Clinical Characteristics

	Total (N = 326)	Women (n = 303)	Men (n = 23)	*P* Value
Age, y, mean (SD)	53.27 (11.09)	53.48 (11.24)	50.48 (8.69)	0.211
Ethnic, race White, n (%)	275 (84.4)	260 (85.8)	15 (65.2)	**0.016**
Employed, n (%)	218 (66.9)	198 (65.3)	20 (87.0)	**0.034**
Education, postsecondary, n (%)	265 (81.3%)	245 (80.9)	20 (87.0)	0.756
Married/partnership, n (%)	239 (73.3)	218 (71.9)	21 (91.3)	0.129
Time since diagnosis, mean (SD)	15.92 (12.47)	16.01 (12.34)	14.78 (14.38)	0.651
SCAD classification type II, n (%)	157 (48.2)	148 (48.8)	9 (39.1)	0.369
Clinical presentation, n (%)				**0.008**
STEMI	97 (30.6)	89 (30.3)	8 (34.8)	
NSTEMI	206 (65.0)	194 (66.0)	12 (52.2)	
Ventricular arrhythmia	4 (1.3)	2 (0.7)	2 (8.7)	
Other	10 (3.2)	9 (3.1)	1 (4.3)	
Initial management, n (%)				0.845
POBA	13 (4.0)	12 (4.0)	1 (4.3)	
DES	25 (7.8)	23 (7.7)	2 (8.7)	
CABG	3 (0.9)	3 (1.0)	0	
Conservative	266 (82.9)	246 (82.6)	20 (87.0)	
Left ventricular ejection fraction, mean (SD)	54.94 (8.69)	55.07 (8.84)	53.33 (6.50)	0.378
Heart rate, beats/min, mean (SD)	68.83 (13.03)	69.22 (13.13)	63.60 (10.48)	0.211
Coronary artery disease, n (%)	91 (27.9)	79 (26.1)	12 (52.2)	**0.007**
Hypertension, n (%)	126 (38.7)	119 (39.3)	7 (30.4)	0.401
Diabetes II, n (%)	18 (5.5)	16 (5.3)	2 (8.7)	0.368
Mental health condition^a^	90 (27.6)	89 (29.4)	1 (4.3)	**0.011**
Self-reported pre SCAD major depression, n (%)	21 (6.4)	21 (6.9)	0 (0)	0.381
Self-reported pre SCAD generalized anxiety disorder, n (%)	46 (14.1)	44 (14.5)	2 (8.7)	0.754
Self-reported pre SCAD PTSD, n (%)	22 (6.7)	22 (7.3)	0 (0)	0.384

**Bolded***P* values indicates statistical significance (*P* < 0.05).

CABG = coronary artery bypass grafting; DES = drug eluting stent; NSTEMI = non–ST-segment elevation myocardial infarction; POBA = plain old balloon angioplasty; PTSD = posttraumatic stress disorder; SCAD = spontaneous coronary artery dissection; STEMI = ST-segment elevation myocardial infarction.

a Clinically significant anxiety or depressive symptoms (GAD-7 or PHQ-9 score ≥10).

### Preferences for intervention content

Intervention preferences are summarized in the Central Illustration and Table 2, ordered from the highest to lowest ratings. The most highly endorsed component was stress management, with 64.5% of participants rating it as very or extremely important (*M* = 3.73 ± 1.16). Relaxation exercises (55.4%, *M* = 3.50 ± 1.21), cognitive-behavioral techniques (56.8%; *M* = 3.45 ± 1.26), and dealing with the trauma of the cardiac event (53.5%; *M* = 3.41 ± 1.36) followed. Topics with moderate perceived importance included positive psychology, such as practicing optimism and identifying personal strengths (48.8%; *M* = 3.31 ± 1.28), and health advocacy training (50.7%; *M* = 3.25 ± 1.40). In contrast, the lowest-rated components were vocational counseling (33.4%; *M* = 2.75 ± 1.40), pharmacological treatments (31.6%; *M* = 2.70 ± 1.31), patient partnering/buddy system (21.4%; *M* = 2.46 ± 1.27), and instrumental support (23.4%; *M* = 2.40 ± 1.29).

**Central Illustration:**
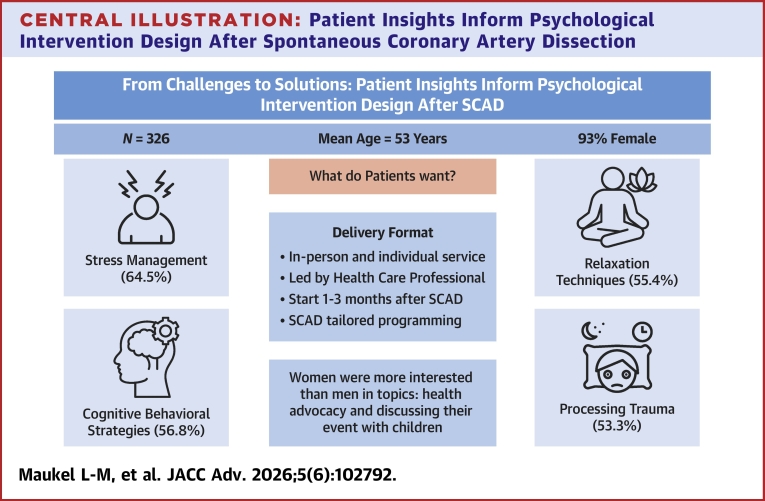
**Patient Insights Inform Psychological Intervention Design After Spontaneous Coronary Artery Dissection** Among 326 participants (93% female; mean age 53 years) top-rated intervention components included stress management (64.5%), relaxation techniques (55.4%), cognitive behavioral strategies (56.8%), and processing the SCAD-related trauma (53.5%). Patients preferred SCAD-specific programming delivered by health care professional, offered within 1 to 3 months postevent. Women expressed greater interest than men in topics such as health advocacy and communication with children. SCAD = spontaneous coronary artery dissection.

**Table 2:** Patient Preferences Regarding Potential Mental Health Intervention Content

Potential Intervention Content	N	Total M (SD)	Highly Important, n (%)	Women M (SD)	Men M (SD)	*P* Value
Stress management	315	3.73 (1.16)	200 (64.5)	3.77 (1.15)	3.30 (1.22)	0.066
Relaxation exercises (eg, deep breathing, mindfulness meditation)	314	3.50 (1.21)	181 (55.4)	3.53 (1.21)	3.05 (1.13)	0.070
Cognitive-behavioral techniques (ie, addressing your thoughts, emotions, and behaviors)	315	3.45 (1.26)	179 (56.8)	3.48 (1.27)	3.09 (1.12)	0.152
Dealing with the trauma of the cardiac event	314	3.41 (1.36)	168 (53.5)	3.45 (1.36)	2.91 (1.35)	0.069
Anxiety treatment (generalized)	314	3.39 (1.42)	174 (55.4)	3.42 (1.42)	3.04 (1.40)	0.226
Sleep hygiene (ie, basic tips to improve your sleep)	312	3.38 (1.31)	171 (54.8)	3.40 (1.30)	3.13 (1.52)	0.335
Self-awareness and self-compassion	310	3.37 (1.28)	166 (53.5)	3.40 (1.27)	2.96 (1.36)	0.110
Tolerating uncertainty	312	3.36 (1.31)	159 (50.9)	3.41 (1.31)	2.74 (1.21)	**0.018∗**
Pacing skills (eg, learn ways to not overdo it)	311	3.33 (1.27)	160 (51.5)	3.36 (1.27)	2.87 (1.22)	0.073
Exercise for mood management	313	3.33 (1.26)	161 (51.4)	3.34 (1.27)	3.17 (1.19)	0.549
Positive psychology (eg, enhancing a sense of belonging, optimism, happiness, gratitude)	311	3.31 (1.28)	152 (48.8)	3.35 (1.27)	2.74 (1.32)	**0.027∗**
Information support sessions from a medical professional (eg, webinars, information sessions)	309	3.29 (1.23)	142 (45.9)	3.32 (1.21)	3.00 (1.35)	0.231
Health advocacy (eg, learning ways to get your needs met in the health care system)	310	3.25 (1.40)	157 (50.7)	3.31 (1.38)	2.61 (1.50)	**0.021∗**
Reassessing values and time management	311	3.24 (1.27)	149 (47.9)	3.27 (1.25)	2.78 (1.35)	0.073
Information support sessions from a mental health professional (eg, webinars, information sessions)	308	3.20 (1.31)	137 (44.5)	3.24 (1.30)	2.65 (1.40)	**0.038∗**
Family functioning (eg, how to manage your family after your cardiac event, how to ask for the support your need, how to cope with the changes in your family).	312	3.19 (1.36)	149 (47.7)	3.21 (1.35)	2.83 (1.50)	0.188
Preventative health behaviors (eg, diet, smoking)	314	3.17 (1.38)	154 (49.0)	3.20 (1.37)	2.78 (1.48)	0.163
Information support materials—guidebooks	311	3.16 (1.22)	135 (43.3)	3.18 (1.22)	2.96 (1.26)	0.398
Behavioral strategies (eg, setting and refining goals)	308	3.15 (1.27)	134 (43.5)	3.17 (1.26)	2.91 (1.38)	0.346
Assertiveness and communication training (eg, saying no, communicating your needs)	309	3.14 (1.34)	149 (48.2)	3.16 (1.34)	2.96 (1.40)	0.491
Resilience-training	307	3.12 (1.30)	133 (43.3)	3.15 (1.30)	2.74 (3.15)	0.140
Depression treatment	314	3.10 (1.44)	149 (47.5)	3.15 (1.44)	2.52 (1.38)	**0.045∗**
Peer support programming	312	3.07 (1.31)	130 (41.6)	3.10 (1.31)	2.70 (1.29)	0.155
Insomnia treatment	309	2.98 (1.46)	138 (44.6)	3.01 (1.45)	2.96 (1.26)	0.158
Relationship functioning (eg, how to manage your relationship since your cardiac event, how to talk to your partner about your cardiac event, your feelings, or needs).	303	2.97 (1.44)	131 (43.2)	2.99 (1.45)	2.77 (1.45)	0.499
How to speak to your children about your cardiac event	307	2.97 (1.42)	132 (43.0)	3.03 (1.40)	2.26 (1.51)	**0.013∗**
Enhancing social support	311	2.96 (1.31)	123 (39.5)	2.99 (1.29)	2.61 (1.53)	0.184
Existential/spiritual approaches (eg, meaning of life/death, human potential/limitations, purpose)	310	2.81 (1.33)	100 (32.2)	2.83 (1.31)	2.52 (1.53)	0.287
Vocational counseling (eg, assistance with return to work or sick/disability forms)^a^	207	2.75 (1.40)	69 (33.4)	2.80 (1.40)	2.30 (1.26)	0.129
Pharmaceuticals (ie, medications to help with mood, anxiety, or sleep)	313	2.70 (1.31)	99 (31.6)	2.73 (1.31)	2.35 (1.27)	0.176
Patient partnering (buddy system) meetings	308	2.46 (1.27)	66 (21.4)	2.47 (1.27)	2.30 (1.22)	0.547
Instrumental support (eg, help with finances, housing, etc.)	312	2.40 (1.29)	73 (23.4)	2.43 (1.29)	2.00 (1.17)	0.121

The mean score (M) refers to the average Likert Scale rating, ranging from 1 to 5, Highly important *N* (%) is calculated as the sum of "very important" and "extremely important" responses. Reported *P* values reflect comparisons of mean values between female and male participants. ∗∗∗*P* < .001, ∗∗*P* < .01, ∗*P* < .05.

a Employed participants (including those on leave).

Significant sex differences were found in several content areas (Table 2). Women rated the following as more important than men: tolerating uncertainty (*M* = 3.41 ± 1.31 vs 2.74 ± 1.21; *P* = 0.018); positive psychology (*M* = 3.35 ± 1.27 vs 2.74 ± 1.32; *P* = 0.027); health advocacy training (*M* = 3.31 ± 1.38 vs 2.61 ± 1.50; *P* = 0.021); information support sessions from a mental health professional (*M* = 3.24 ± 1.30 vs 2.65 ± 1.40; *P* = 0.038); depression treatment (*M* = 3.15 ± 1.44 vs 2.52 ± 1.38; *P* = 0.045); and how to speak to children about one’s cardiac event (*M* = 3.03 ± 1.40 vs 2.26 ± 1.51; *P* = 0.013). No topics were rated significantly higher by men compared to women. Preferred intervention content did not differ across categories of time since diagnosis.

### Preferences for intervention delivery

Patients’ preferred delivery formats are presented in Table 3. Participants expressed stronger preferences for in-person programming (43.9%; *M* = 3.06 ± 1.31), followed by webinars (37.1%; *M* = 2.92 ± 1.24), interactive online sessions (36.0%; *M* = 2.87 ± 1.34), self-guided materials (37.1%; *M* = 2.82 ± 1.22), and phone-based interventions (27.5%; *M* = 2.69 ± 1.21). Individual interventions were preferred (46.2%; *M* = 3.18 ± 1.27) over group (27.9%; *M* = 2.58 ± 1.29), couples-based (19.6%; *M* = 2.20 ± 1.26) and family-based interventions (15.4%; *M* = 2.17 ± 1.20). Programs facilitated by health care professionals were rated the highest (56.2%; *M* = 3.41 ± 1.13), followed by joint facilitation with peers (52.4%; *M* = 3.30 ± 1.26), and peer-led interventions (24.4%; *M* = 2.51 ± 1.22). Locally inclusive programming (47.2%; *M* = 3.18 ± 1.24) was preferred over provincial (34.4%; *M* = 2.87 ± 1.23) or national offerings (32.7%; *M* = 2.81 ± 1.24). SCAD-specific programs received the strongest interest (70.8%; *M* = 3.85 ± 1.12), followed by women-specific (52.4%; *M* = 3.31 ± 1.35) and programs for younger individuals (23.6%; *M* = 2.36 ± 1.38). Participants expressed the highest interest in interventions starting 1 to 3 months postevent (61.8%; *M* = 3.49 ± 1.40), followed closely by 3 to 6 months (55.9%; *M* = 3.38 ± 1.38), 6 to 12 months (48.8%; *M* = 3.21 ± 1.37), and 12+ months (47.8%; *M* = 3.14 ± 1.33). Patients endorsed monthly (45.2%; *M* = 3.18 ± 1.22) rather than weekly (32.9%, *M* = 2.73 ± 1.37) sessions. Evening sessions were rated the highest (43.3%; *M* = 3.09 ± 1.33), followed by daytime (32.8%, *M* = 2.69 ± 1.32) and weekend sessions (23.6%, *M* = 2.28 ± 1.34).

**Table 3:** Patient Preferences Regarding the Intervention Modality

	N	Total M (SD)	Highly Interested, n (%)	Women M (SD)	Men M (SD)	*P* Value
Delivery format						
In-person programming	312	3.06 (1.31)	137 (43.9)	3.08 (1.31)	2.91 (1.31)	0.567
Webinars	310	2.92 (1.24)	115 (37.1)	2.97 (1.23)	2.30 (1.15)	**0.012∗**
Online programming with participant interaction (e.g., Zoom)	311	2.87 (1.34)	112 (36.0)	2.89 (1.34)	2.70 (1.33)	0.506
Self-guided programs (e.g., workbooks)	311	2.82 (1.22)	115 (37.1)	2.86 (1.22)	2.30 (1.15)	**0.034∗**
Phone-based interventions	309	2.69 (1.21)	85 (27.5)	2.71 (1.21)	2.41 (1.22)	0.266
Participant format						
Individual	307	3.18 (1.27)	142 (46.2)	3.19 (1.26)	3.04 (1.33)	0.586
Group	311	2.58 (1.29)	87 (27.9)	2.60 (1.31)	2.26 (1.05)	0.220
Couple	301	2.20 (1.26)	59 (19.6)	2.21 (1.27)	2.17 (1.11)	0.910
Family	304	2.17 (1.20)	47 (15.4)	2.19 (1.21)	1.96 (1.02)	0.374
Facilitator type						
Health care professional facilitated	308	3.41 (1.13)	173 (56.2)	3.43 (1.13)	3.17 (1.11)	0.300
Patient and health care professional facilitated	307	3.30 (1.26)	161 (52.4)	3.32 (1.26)	3.13 (1.29)	0.496
Patient (peer) facilitated	307	2.51 (1.22)	75 (24.4)	2.52 (1.22)	2.35 (1.27)	0.514
Geographic reach						
Locally inclusive	305	3.18 (1.24)	144 (47.2)	3.22 (1.24)	2.74 (1.14)	0.075
Provincially inclusive	305	2.87 (1.23)	105 (34.4)	2.91 (1.23)	2.35 (1.07)	**0.034∗**
Canada-wide inclusive	303	2.81 (1.24)	99 (32.7)	2.84 (1.24)	2.48 (1.20)	0.183
Target population						
Specific to the cardiac diagnosis	308	3.85 (1.12)	218 (70.8)	3.89 (1.12)	3.35 (1.03)	**0.025∗**
Women specific	305	3.31 (1.35)	160 (52.4)	3.43 (1.28)	1.77 (1.23)	**<0.001∗∗∗**
Younger populations specific	296	2.36 (1.38)	70 (23.6)	2.39 (1.40)	2.00 (1.11)	0.129
Timing						
1-3 months postevent	298	3.49 (1.40)	184 (61.8)	3.51 (1.42)	3.22 (1.13)	
3-6 months postevent	297	3.38 (1.38)	166 (55.9)	3.43 (1.39)	2.77 (1.15)	**0.033∗**
6-12 months postevent	297	3.21 (1.37)	145 (48.8)	3.27 (1.37)	2.48 (1.20)	**0.008∗∗**
12 months + postevent	299	3.14 (1.33)	143 (47.8)	3.18 (1.33)	2.61 (1.20)	**0.046∗**
Beginning in hospital	300	3.08 (1.45)	133 (44.4)	3.11 (1.47)	2.70 (1.22)	0.188
Session frequency						
Once per month	301	3.18 (1.22)	136 (45.2)	3.21 (1.23)	2.86 (1.04)	0.204
Once per week	289	2.73 (1.37)	95 (32.9)	2.73 (1.39)	2.77 (1.23)	0.880
Session timing						
Evening	300	3.09 (1.33)	130 (43.3)	3.09 (1.35)	3.09 (0.95)	0.999
Daytime	302	2.69 (1.32)	99 (32.8)	2.73 (1.34)	2.17 (0.98)	**0.016∗**
Weekend	293	2.28 (1.34)	69 (23.6)	2.24 (1.34)	2.65 (1.30)	0.163

The mean score (*M*) refers to the average Likert Scale rating, ranging from 1 to 5, highly interested *N* (%) is calculated as the sum of "very interested" and "extremely interested" responses. Reported *P* values reflect comparisons of mean values between female and male participants.

Women and men showed similar preferences for in-person and individual interventions (Table 3). However, women rated diagnosis-specific content (*M* = 3.89 ± 1.12 vs 3.35 ± 1.03, *P* = 0.025) and women-specific programming (*M* = 3.43 ± 1.28 vs 1.77 ± 1.23, *P* < 0.001) significantly higher than men. Regarding session timing, women gave significantly higher ratings for daytime sessions compared to men (*M* = 2.73 ± 1.34 vs 2.17 ± 0.98, *P* = 0.016).

### Results from exploratory analyses

The highest preferences for intervention content did not significantly differ based on time since diagnosis.

Patients with pregnancy-related SCAD had higher preferences for intervention content on dealing with the trauma of the cardiac event (*M* = 4.20 ± 1.01 vs 3.38 ± 1.38; *P* = 0.003), exercise for mood management (*M* = 3.80 ± 0.78 vs 3.30 ± 1.28; *P* = 0.020), and preventative health behaviors (*M* = 4.07 ± 0.88 vs 3.11 ± 1.38; *P* < 0.001).

In multiple regression analyses, female sex was significantly associated with preference for content focused on speaking to children about the event (*B* = 0.71; *SE* = 0.31; *P* = 0.022). Trends were observed for preferences related to (*B* = 0.51; *SE* = 0.28; *P* = 0.068), positive psychology (*B* = 0.46; *SE* = 0.27; *P* = 0.089), and health advocacy (*B* = 0.54; *SE* = 0.30; *P* = 0.068).

### Open-ended responses

Several participants expressed a strong desire for increased direct contact with a cardiologist or SCAD specialist, noting that some had never received a referral. Many participants requested SCAD-specific guidelines for physical activity, as exercise had served as a key coping strategy before their SCAD event.

## Discussion

This study moves the field from understanding *what* the psychological challenges of SCAD are to understanding *how* patients want them addressed. This large quantitative investigation provides new insights into survivors’ preferences for psychological intervention content and format. Examining sex differences further clarified how preferences differ between female and male survivors.

In this study, we identified a demand for interventions targeting stress management, relaxation techniques, cognitive-behavioral strategies, and trauma processing related to the SCAD event. These findings go beyond earlier qualitative reports that emphasized a general desire for psychosocial support,^39^, ^40^, ^41^ by revealing a clear preference for structured, evidence-based therapeutic interventions. Preliminary work has implemented some of these intervention strategies—including mindfulness-based relaxation techniques, cognitive-behavioral therapy, and third-wave cognitive-behavioral therapy approaches such as acceptance and commitment therapy.^18^, ^19^, ^20^ None of these proof-of-concept studies, however, demonstrated clinically significant improvements in mental health outcomes, indicating that further revision is required. Our findings may guide these revisions.

Postintervention feedback from 1 program highlighted the benefits of skills development, although some participants found the mindfulness components challenging. Another program reported notably high attrition after the first session as participants indicated that listening to others share their SCAD experiences was anxiety-provoking,^20^ suggesting a potential risk of retraumatization. Incorporating trauma-focused care, specifically called for by our participants, should be a key component of future interventions.

The 3 initial SCAD-specific interventions were delivered in group format where participants reported benefits interacting with peers,^18^, ^19^, ^20^ but also challenges related to oversharing.^20^ Although our participants acknowledged the value of peer support, they rated the group format as less appealing—possibly because patient-led peer support groups and organizations such as Women@Heart^42^ and Beat SCAD^43^ already exist. Our patient partners highlight that some patients prefer the safety and expertise of professionals alone and view peer support as a complement to rather than replacement of professional supports. This individualized format may reflect a broader desire for personalized and direct care, commonly seen in cardiac populations.^44^

Although our patients prefer in person encounters, past research reported requests for home-based programs due to the flexibility, convenience, and independence they afford.^44^ With growing acceptance of virtual care among both patients and providers, especially in geographically expansive countries like Canada,^45^^,^^46^ a hybrid model combining in-person and virtual sessions is increasingly promising. In-person interactions allow for richer nonverbal communication and stronger rapport-building,^47^ whereas virtual options enhance adherence by offering flexibility for logistical and scheduling challenges, particularly for those in remote areas or with caregiving responsibilities.^48^^,^^49^ Notably, video-based psychological interventions have demonstrated effectiveness comparable to face-to-face care in both mental health and cardiac populations.^50^^,^^51^

In contrast to the broader CR population who are more ambivalent about psychological care and more likely to rely on passive recovery over time,^48^^,^^49^ there is a clear demand for early, individualized, SCAD-specific psychological interventions. That said, our participants with SCAD reported a lower perceived need for psychological care during their hospital stay, possibly due to a focus on physical health and CR. Previous qualitative data suggest that patients desire accessible SCAD-related information immediately after the event.^41^ This points to the value of a stepped approach of providing educational materials during hospitalization, followed by structured psychological interventions. Patients in this study indicated a preference for initiating interventions 1 to 3 months postdiagnosis, a timeframe that aligns with previous research showing improved mortality outcomes when psychological treatment begins within 2 months after a cardiac event, rather than early or substantially delayed.^52^^,^^53^

Participants expressed a preference for monthly rather than weekly sessions, likely reflecting the complex demands of their lives as SCAD survivors (eg, young women with family responsibilities and work commitments).^10^ Although monthly sessions may be more feasible, they may not offer sufficient frequency to achieve optimal outcomes. Psychological interventions typically yield better results when delivered weekly, as this allows for consistent support and ongoing emotional and cognitive processing.^54^^,^^55^

We observed significant sex differences in intervention preferences. Female participants reported a greater need for support in managing the uncertainty related to their diagnosis, and expressed a stronger desire for depression treatment, consistent with previous qualitative findings.^48^ Our previous research showed that the psychological burden expressed by female SCAD survivors was 2 to 5 times greater than males,^12^ highlighting the need to enhance CR among this group. Women also expressed a greater interest in content related to health advocacy, such as learning how to navigate the health care system and effectively communicate their needs. Previous research indicates that women with cardiac events often feel less informed and less satisfied with the information provided, which could hinder adherence.^56^^,^^57^ Female participants also want support for communicating with their children about their cardiac event. This desire likely reflects the demographic profile of SCAD patients—predominantly middle-aged women, more than half of whom live with children—as well as gendered caregiving norms. Women often serve as primary caregivers^58^ and emotional facilitators within families, potentially increasing their need for guidance when discussing their cardiac events with family members. Female participants expressed interest in both daytime and evening sessions, underscoring how balancing work, caregiving, and rehabilitation could shape participation in their recovery. Flexible scheduling is the key to improving accessibility and engagement, especially for women managing multiple roles.^59^^,^^60^ Not surprisingly, over half of the female participants (55.1%) expressed highest interest in women-specific interventions, aligning with qualitative findings that reported a feeling of isolation during recovery, particularly in male-dominated CR classes.^48^ One approach could involve developing optional modules on gender-sensitive topics that emerged in this study, such as health advocacy and communicating with children about the SCAD event. These modules could be made available to all patients, regardless of sex assigned at birth, allowing individuals to select content relevant to their personal experience. It may also be appropriate to offer sex-specific modules, for example, for pregnancy-related SCAD, given that these patients are generally younger and reported greater interest in trauma-focused content.

Taken together, our findings provide patient-guided steps for psychological intervention development tailored for SCAD survivors. Aligning interventions with patient preferences is critical for adherence: evidence suggests that individuals who receive their preferred form of care are more likely to engage meaningfully and less likely to discontinue treatment.^61^ This point is especially relevant given the high dropout rates in preliminary SCAD-specific psychological interventions.^18^, ^19^, ^20^ Following the obesity-related behavioral intervention trials model for psychological and behavioral intervention development, future directions include the development of sex- and gender-sensitive psychological intervention in collaboration with SCAD survivors, and mental health and cardiology experts. A codesign approach will help ensure that the intervention is not only clinically relevant and feasible, but also responsive to the lived experiences and patient-defined priorities.^33^ Next steps will involve manual development and phase IIa feasibility testing to evaluate acceptability and preliminary outcomes,^34^ with intervention materials which can be adapted to various settings and modes of delivery (eg, group or individual, online or in person). Such adaptability may support dissemination beyond specialized CR and private clinics while preserving core intervention components.

### Study limitations

Although the findings offer valuable insights, there are some limitations. Consistent with previous SCAD studies,^62^ the sample was predominantly White (85%), and highly educated, which may limit generalizability to more diverse SCAD populations. Accordingly, the results should be interpreted as hypothesis-generating rather than representing universal preferences across all SCAD survivors. This highlights the need for more inclusive recruitment strategies to ensure the perspectives and needs of diverse populations are adequately represented in future research.^63^ Although our sample reflects the clinical reality of SCAD, with approximately 90% of cases occurring in women, the inclusion of a larger male sample would improve the generalizability of the findings.

While a brief example was provided for relaxation techniques, no specific description was given for stress management; thus, participants may have interpreted these strategies as overlapping.

The cross-sectional design of the study limits our ability to assess how preferences for psychological interventions evolve over time. Longitudinal prospective studies could help identify these evolving needs and optimize the timing and content of interventions. However, in a post hoc analysis, the highest preferences for intervention content and delivery modalities did not significantly differ based on time since diagnosis.

Other variables, including mental health status and physical burden, may influence intervention preferences; however, in exploratory analyses adjusting for these factors, the significant association between female sex and greater preference for content related to communicating with children about the SCAD event was retained. Future studies should nevertheless incorporate in-hospital clinical severity indicators (eg, infarct size, resuscitation events, complications, and intensive care unit stay), as these variables were not available in the current data set and may further influence psychological trajectories and intervention needs. In addition, 3 trends toward higher preference scores were observed for content addressing tolerating uncertainty, health advocacy, and positive psychology, although these did not reach statistical significance. Given the small male subgroup (n = 23) and limited power of adjusted analyses, findings should be interpreted as hypothesis generating. Nevertheless, they support the potential importance of considering sex and gender in the design and tailoring of intervention content.

## Conclusions

Patients with SCAD demonstrate a clear preference for early, individualized, SCAD-specific psychological interventions that prioritize cognitive and emotional processing that prioritize cognitive and emotional processing, ideally delivered by health care professionals. The study also highlights significant sex differences in intervention preferences, suggesting the need for sex- and gender-sensitive approaches to care. To address these diverse needs, it is crucial to develop and test tailored psychological interventions in collaboration with SCAD survivors. This collaborative, patient-centered approach represents an important next step toward improving psychological outcomes and ensuring that interventions are both relevant and effective for this patient population.

Perspectives **COMPETENCY IN PATIENT CARE AND PROCEDURAL SKILLS:** Psychological distress is associated with worse cardiovascular outcomes, and patients with SCAD experience particularly high levels of distress that are insufficiently addressed by the standard care. This study identifies patient-prioritized targets for psychological care, indicating that SCAD survivors value early, SCAD-specific interventions focusing on stress management, cognitive-behavioral strategies, and emotional and trauma-related processing. Clinicians should recognize these unmet needs, discuss psychological concerns early after the acute event, and refer to SCAD-specific resources. Patient preferences should be integrated into tailored psychological interventions. Incorporating sex- and gender-sensitive content—particularly for women—may enhance patient engagement, relevance, and overall quality of care.

**TRANSLATIONAL OUTLOOK:** Moving from understanding the recovery challenges faced by patients with SCAD to how best to support SCAD survivors, this study summarizes specific intervention approaches identified by over 300 patients and guides intervention development in collaboration with patients and providers. Future research should codevelop and test tailored interventions with SCAD survivors, evaluating feasibility, efficacy, and potential impacts on cardiovascular markers and adverse events in controlled trials. Incorporating sex- and gender-sensitive content may enhance engagement and clinical relevance. Integrating evidence-informed, patient-centered psychological interventions into cardiac care pathways has the potential to address an important unmet need and improve long-term psychosocial and cardiac outcomes in this population.

## Funding support and author disclosures

This work was supported by a 10.13039/501100000024Canadian Institutes of Health Research (CIHR) Project Grant Program (180593, PI: Tulloch). Dr Madan is supported by the Heart & Stroke Foundation Polo Chair in Cardiology at the University of Toronto. All other authors have reported that they have no relationships relevant to the contents of this paper to disclose.
